# Functional abdominal pain disorders in adolescents in Indonesia and their association with family related stress

**DOI:** 10.1186/s12887-019-1682-5

**Published:** 2019-09-26

**Authors:** Hanifah Oswari, Fatima Safira Alatas, Badriul Hegar, William Cheng, Arnesya Pramadyani, Marc Alexander Benninga, Shaman Rajindrajith

**Affiliations:** 1 Department of Child Health, Gastrohepatology division, Cipto Mangunkusumo Hospital, Faculty of Medicine, Universitas Indonesia, Jl. Salemba 6, Jakarta, 10430 Indonesia; 2 Department of Paediatric Gastroenterology and Nutrition, Emma Children’s, Hospital, Academic Medical Centre, Amsterdam, The Netherlands; 3 Department of Paediatrics, University of Kelaniya, Ragama, Sri Lanka

**Keywords:** Abdominal pain, Functional gastrointestinal disorder, Adolescent, Emotional stress

## Abstract

**Background:**

Functional abdominal pain disorders (FAPD) have been widely reported as a major group of gastrointestinal disorders across the world. This study describes the prevalence, related factors, symptomatology and its relationship to emotional stress in Indonesian adolescents.

**Methods:**

This is a cross-sectional study. Adolescents aged 10 to 17 years from nine randomly selected state schools from five districts of Jakarta, Indonesia, were invited to participate. A translated and validated Rome-III self-administered-questionnaire was used to collect data on gastrointestinal symptoms. Data on sociodemographic characteristics, intestinal as well as extra-intestinal symptoms, and exposure to stressful life events were also collected using a separate validated questionnaire.

**Results:**

A total of 1813 questionnaires were included in the analysis [males 739 (40.8%) mean age of 13.54 years + 0.89]. Of them, 209 children (11.5%) fulfilled Rome III criteria of FAPD. Functional abdominal pain (FAP) was reported as the most prevalent subtype (5.8%), followed by functional dyspepsia (3.3%), irritable bowel syndrome (2%) and abdominal migraine (0.4%). The prevalence was higher in girls (*p* < 0.05) and those exposed to family-related stressful life events (*p* < 0.05). They include divorce or separation of parents (adjusted OR 2.55, 95% CI 1.75–3.7, *p* = < 0.001), death of a close family member (adjusted OR 2.24, 95% CI 1.39–3.59, *P* = 0.001), and father’s alcoholism (adjusted OR 1.94, 95% CI 1.22–3.1, *P* = 0.005).

**Conclusions:**

FAPD are common paediatric entities among Indonesian adolescents with a prevalence of 11.5%. FAPD were noted to be higher in girls and adolescents exposed to family-related stressful life events.

## Background

Functional abdominal pain disorders (FAPD) are a common problem in paediatric practice. This group consists of functional abdominal pain (FAP), irritable bowel syndrome (IBS), functional dyspepsia (FD) and abdominal migraine (AM). They are characterised by the absence of identifiable organic causes for the symptoms [1–3]. Reported prevalence of these disorders vary from study to study [1, 4, 5]. A recent meta-analysis showed 13.5% of children are suffering from FAPD across the world [2]. In addition, this group of disorders poses a significant strain on the global healthcare system due to sheer numbers and recurring healthcare costs [6–8]. Furthermore, FAPD are significantly associated with lower quality of life and are ranked as second in causing school absence [2, 9, 10]. Children who suffer from FAPD are shown to develop high rates of anxiety disorders during adolescence and young adulthood [3].

Factors related to FAPD may vary according to region and age group. A previous study performed in South East Asia, with a prevalence rate of FAPD of 10.2%, showed that children from lower income families, lower educational background of father and studying in a rural school had significant relationships with FAPD [11]. A systematic review and meta-analysis regarding epidemiology of FAPD concluded that the prevalence of FAPD are affected by female gender, psychological disorders, stress and traumatic life events [2].

There are approximately 50 million (18%) adolescents in Indonesia, the fourth most populated country in the world. However, no data are available to describe the prevalence of FAPD [12]. Therefore, we conducted the first epidemiological study to report on the prevalence of overall FAPD and to describe different types of FAPD, along with social and psychological factors related to these disorders.

## Methods

### Study design and sample

This was a cross-sectional survey conducted in nine randomly selected state Junior High Schools from five administrative cities (municipalities) of Jakarta, Indonesia; (Central, North, West, South Jakarta, and East Jakarta). Jakarta is the capital city of Indonesia and also the biggest metropolitan city in the country in which all cities are classified as urban areas. This study refers to a previous study of FAPD conducted in Sri Lanka, which showed a prevalence of FAPD of 12.5% [1]. Considering that Indonesia has some similar characteristics with Sri Lanka as a developing country in the South Asian region, the expected prevalence of FAPD in this study was 12.5%. With 80% of power, 5% significance and 20% of attrition, minimum sample size calculated for each study site in this study was 172.

### Data collection

Data were collected between July and December 2016. From each municipality, two schools were randomly selected. From every school, 6 to 8 classes were randomly selected from 7th to 9th grade students which included adolescents between 10 to 17 years of age. School authorities and parents were informed regarding the study and informed written consent from the parents and assent forms from the students were gathered before the initiation of the study.

Data were collected using a pre-tested questionnaire that consisted of 2 parts. Part 1 included questions on socio-demographic and family factors and exposure to stressful live events. Part 2 included the Questionnaire on Paediatric Gastrointestinal Symptoms Rome III version from previous studies, self-reported form for children and adolescents (10 years of age and older) [1], translated into the native language for Indonesian children. As part of the questionnaire validation process, forward translation was performed by two medical translators, followed by a reconciliation stage. Backward translations into the original language were then produced by two native English translators, also followed by further reconciliation where misunderstandings or unclear wordings in the initial translations were discussed. Subsequently the final questionnaire was validated in a small sample before being used in the study.

Besides the data completion, the inclusion criteria in this study are children with 10–17 years of age, and generally in good health condition. Selection bias was talked in this study by providing large amount of samples. Measurement and confounding bias was controlled before from the validation of pretested questionnaire used in this study. Ethical approval for the present study was granted by the Medical Ethics Committee of the Universitas Indonesia.

There were 2 rounds for collecting the questionnaires with different methods: take-home test (July to August 2016) and examination setting (November–December 2016). In the first round, questionnaires distributed for this study were filled in the student’s home after explanation from the research team. However, with low return rates, we conducted a second round from different schools but in the same municipal areas as the first round with examination setting in which students filled the questionnaires in front of the study team.

### Diagnosis

Children with abdominal pain were categorised into FAPD (FD, IBS, AM, and FAP) using Rome III criteria [13].

### Data analysis

Analysis of data was performed using SPSS 20 (IBM Corp. Armonk, NY, USA). The χ^2^ and Fisher exact tests with at least *p* < 0.05 was taken as significant for bivariate analysis. Multivariate analysis with logistic regression were attempted for bivariate *p*-value below 0.2 to obtain adjusted Odds Ratios for socio-demographic factors and stressful life events.

## Results

Three thousand five hundred and seventy two (3572) questionnaires were distributed for this study. In the first round, 2718 questionnaires were distributed and were filled at student’s home after explanation from the research team. Of them, only 1196 (44%) were returned and 1012 questionnaires (37.23%) had all the compulsory data to be taken into account for analysis. In the second round, 854 questionnaires were assigned in an examination setting. There were 811 (95%) questionnaires collected with this method and 801 (93.79%) were eligible to be included in the analysis.

Therefore, 1813 participants were included in the analysis [739 (40,76%) boys]. The participants were 10 to 17 years of age with a mean age of 13.54 years, SD 0.89 years.

### Prevalence of FAPD

Based on Rome III criteria classification, 209 children were diagnosed with at least 1 FAPD (Table 1), providing an overall prevalence of 11.5%. FAP (5.8%) was the most common FAPD followed by functional dyspepsia (3.3%), irritable bowel syndrome (2%) and abdominal migraine (0.4%).

**Table 1:** Prevalence of functional abdominal pain disorders according to sex

FAPD type	Male, n (%, 95 CI)	Female, n (%; 95% CI)	Total, n (%; 95% CI)
FD	16 (2.2; 1.2 to 3.5)	44 (4.1; 3 to 5.5)	60 (3.3; 2.5 to 4.2)
IBS	15 (2; 1.1 to 3.3)	22 (2; 1.3 to 3.1)^a^	37 (2; 1.4 to 2.8)
AM	1 (0.1; 0 to 0.8)	7 (0.7; 0.3 to 1.3)	8 (0.4; 0.2 to 0.9)
FAP	34 (4.6; 3.2 to 6.4)	71 (6.6; 5.2 to 8.3)	105 (5.8; 4.7 to 6.9)
Functional abdominal pain disorders -total	66 (8.9; 7 to 11.2)	143 (13.3; 11.3 to 15.5)	209 (11.5; 10 to 13)

*FAPD* functional abdominal pain disorders, *FD* functional dyspepsia, *IBS* irritable bowel syndrome, *AM* abdominal migraine, *FAP* functional abdominal pain. ^a^One also had AM

### Association between socio-demographic characteristics and abdominal pain–predominant FAPD

Table 2 shows the association between the socio-economic characteristics and FAPD. Multivariate analysis found that female gender was significantly associated with FAPD when compared to the male gender (OR 1.57, 95% CI 1.15–2.15, *p* = 0.004). A significantly lower incidence of FAPD was found in children from south Jakarta compared to other area in Jakarta (Table 2).

**Table 2:** Distribution of subjects according to sociodemographic characteristics and prevalence of abdominal pain in each category

Variable		Distribution of subjects		Prevalence of AP-FGID in each category (%; 95% CI)	Crude OR (CI 95%)	p value	Adjusted OR (CI 95%)	Adjusted p value
		Controls *N*=1.604 n(%; 95% CI)	AP-FGID *N*= 209 n(%; 95% CI)					
Age	10-12 y.o	204 (12.7; 11.1 to 14.4)	19 (9.1; 5.6 to 13.8)	8.5 (5.2 to 13)	1.00 (ref)	-		
	13-15 y.o	1382 (86.2; 84.4 to 87.8)	188 (90; 85.1 to 93.7)	12 (10.4 to 13.7)	1.46 (0.89-2.39)	0.13		
	16-17 y.o	18 (1.1; 0.7 to 1.8)	2 (1; 0.1 to 3.4)	10 (1.2 to 31.7)	1.19 (0.26-5.54)	0.82		
					*p* = 0.316			
Gender	Male	673 (42; 39.5 to 44.4)	66 (31.6; 25.3 to 38.3)	8.9 (7 to 11.2)	1.00 (ref)	-	1.00 (ref)	-
	Female	931 (58;55.6 to 60.5)	143 (68.4; 61.7 to 74.7 )	13.3 (11.3 to 15.5)	1.61 (1.16 - 2.23)	0.04	1.57 (1.15-2.15)	0.004
					*p*=0.004		*P*=0.004	
Family size	Only child	122 (7.6; 6.4 to 9)	13 (6.2; 3.4 to 10.4)	9.6 (5.2 to 15.9)	1.00 (ref)	-		
	2-3 children	1172 (73.1; 70.8 to 75.2)	158 (75.6; 69.2 to 81.3)	11.9 (10.2 to 13.7)	1.26 (0.69-2.29)	0.44		
	≥4 children	310 (19.3; 17.4 to 21.3)	38 (18.2; 13.2 to 24.1)	10.9 (7.8 to 14.7)	1.15 (0.59-2.23)	0.68		
					*p*= 0.683			
Birth order^a^	Eldest	558 (37.6; 35.1 to 40.1)	79 (40.3; 33.4 to 47.5)	12.4 (9.9 to 15.2)	1.00 (ref)	-		
	Youngest	528 (38.6; 33.1 to 38.1)	73 (37.2; 30.5 to 44.4)	12.1 (9.6 to 15)	1.26 (0.7-2.3)	0.44		
	Other	398 (26.8; 24.6 to 29.2)	44 (22.4; 16.8 to 28.9)	10 (7.3 to 13.1)16.8 to 28.9	1.15 (0.59-2.23)	0.68		
					*p*= 0.68			
Mother^b^	Employed	441 (27.6; 25.3 to 29.7)	49 (23.4; 17.9 to 29.8)	12.1 (10.4 to 14)	1.00 (ref)	-		
	Housewife	1158 (72.4;69.9 to 74.4)	160 (76.6; 70.2 to 82.1 )	10 (7.5 to 13)	1.24 (0.89-1.74)	0.21		
					*p*=0.21			
Father's employment	Leading profession (eg. Doctor, engineer)	17 (1.1;0.6 to 1.7)	3 (1.4; 0.3 to 4.1)	15 (3.2 to 37.9)	1.00 (ref)	-		
	Lesser profession (eg. Nurse, teacher)	500 (31.2; 28.9 to 33.5)	57 (27.3; 21.4 to 33.8)	10.2 (7.8 to 13.1)	0.65 (0.18-2.27)	0.496		
	Skilled non-manual (eg. Clerk)	688 (42.9; 40.5 to 45.4)	101 (48.3; 41.4 to 55.3)	12.8 (10.5 to 15.3)	0.83 (0.24-2.89)	0.77		
	Skilled manual	239 (14.9; 13.2 to 16.7)	28 (13.4; 9.1 to 18.8)	10.5 (7.1 to 14.8)	0.66 (0.18-2.41)	0.53		
	Unskilled/unemployed	160 (10; 8.6 to 11.5)	20 (9.6; 5.9 to 14.4)	11.1 (6.9 to 16.6)	0.71 (0.19-2.63)	0.61		
					*p*=0.61			
Maternal employment	Leading profession (eg. Doctor, engineer)	14 (0.9; 0.5 to 1.5)	1 (0.5; 0 to 2.6)	6.7 (0.2 to 31.9)	1.00 (ref)	-		
	Lesser profession (eg. Nurse, teacher)	180 (11.2; 9.7 to 12.9)	25 (12; 7.9 to 17.2)	12.2 (0.8 to 17.5)	1.94 (0.24-15.43)	0.53		
	Skilled non-manual (eg. Clerk)	193 (12; 10.5 to 13.7)	17 (8.1; 4.8 to 12.7)	8 (4.8 to 12.6)	1.23 (0.15-9.95)	0.84		
	Skilled manual	18 (1.1; 0.7 to 1.8)	0	0	0	0.99		
	Unskilled/unemployed	1199 (74.8;72.5 to 76.9)	166 (79.4; 73.3 to 84.7)	12.2 (10.5 to 14)	1.94 (0.25-14.84)	0.52		
					*p*=0.511			
Location of school	South Jakarta	410 (25.6; 23.4 to 27.8)	27 (12.9; 8.7 to 18.2)	6.2 (4.1 to 8.9)	1.00 (ref)	-	1.0 (ref)	-
	North Jakarta	188 (11.7; 10.2 to 13,4)	35 (16.7; 12 to 22.5)	15.7 (11.2 to 21.1)	2.83 (1.66-4.8)	<0.001	2.72 (1.59-4.64)	<0.001
	Central Jakarta	302 (18.8; 16.9 to 20.8)	38 (18.2; 13.2 to 24.1)	11.2 (0.8 to 15)	1.91 (1.14-3.2)	0.03	1.64 (0.96-2.79)	0.015
	East Jakarta	366 (22.8; 20.8 to 25)	56 (26.8; 20.9 to 33,3)	13.3 (10.2 to 16.9)	2.32 (1.44-3.76)	0.02	2.08 (1.27-3.38)	0.001
	West Jakarta	338 (21.1; 19.1 to 23.2)	53 (25.4; 19.6 to 31.8)	13.6 (10.3 to 17.4)	2.38 (1.47-3.87)	0.001	2.3 (1.4-3.76)	<0.001
					*p*=0.01		*p*=0.01	

^a^Family with more than one child

^b^Living mother

### Pain characteristics in children with abdominal pain–predominant FAPD

The distributions of pain characteristics of children with FAPD are presented in Table 3. The pain characteristics were not significantly different between the sub-types (*p* > 0.05).

**Table 3:** Abdominal pain characteristics according to the FAPD

	FD, n (%; 95% CI)	IBS, n (%; 95% CI)	AM, n (%; 95% CI)	FAP, n (%; 95% CI)	Total, n (%; 95% CI)
Frequency of pain					
Once per mo	0	0	3 (37.5; 8.5 to 75.5)	0	3 (1.4; 0.3 to 4.1)
Once per wk	27 (45; 32.1 to 58.4)	19 (51.3; 34.4 to 68.1)	1 (12.5; 0.3 to 52.7)	63 (60; 50 to 69.4)	110 (52.6; 45.6 to 59.6)
Several times per wk	32 (53.3; 40 to 66.3)	17 (45.9; 29.5 to 63.1)	4 (50; 15.7 to 84.3)	38 (36.2; 27 to 46.1)	90 (43.1; 36.3 to 50.1)
Everyday	1 (1.7; 0 to 8.9)	1 (2.7; 0.1 to 14.2)	0	4 (3.8; 1 to 9.5)	6 (2.9; 1.1 to 6.1)
Duration of pain					
1 mo or less	2 (3.3; 0.4 to 11.5)	0	6 (75; 34.9 to 96.8)	0	8 (3.8; 1.7 to 7.4)
2 mo	17 (28.3; 17.5 to 41.4)	9 (24.3; 11.8 to 41.2)	0	29 (27.6;19.3 to 27.2)	55 (26.3; 20.5 to 32.8)
3 mo	12 (20; 10.8 to 32.3)	10 (27; 13.8 to 44.1)	2 (25; 3.2 to 65.1)	21 (20; 12.8 to 28.9)	44 (21.1; 15.7 to 27.2)
4–11 mo	12 (20; 10.8 to 32.3)	3 (8.1; 1.7 to 21.9)	0	15 (14.3; 8.1 to 22.5)	30 (14.4; 9.9 to 19.9)
> =12 mo	17 (28.3;17.5 to 41.4)	15 (40.5; 24.8 to 57.9)	0	40 (38.1; 28.8 to 48.1)	72 (34.4; 28 to 41.3)
Duration of a pain episode					
< 1 h	35 (58.3; 44.9 to 70.9)	27 (73; 55.9 to 86.2)	0 (0)	67 (63.8; 53.9 to 73)	129 (61.7; 54.8 to 68.3)
1–2 h	19 (31.7; 20.3 to 45)	5 (13.5; 4.5 to 28.8)	4 (50; 15.7 to 84.3)	25 (23.8; 16 to 33.1)	53 (25.4; 19.6 to 31.8)
3–4 h	5 (8.3; 2.8 to 18.4)	4 (10.8; 3 to 25.4)	2 (25; 3.2 to 65.1)	5 (4.8; 1.6 to 10.8)	15 (7.2; 4.1 to 11.6)
Most of the day	1 (1.7; 0 to 8.9)	1 (2.7; 0.1 to 14.2)	2 (25; 3.2 to 65.1)	8 (7.6; 3.3 to 14.5)	12 (5.7; 3 to 9.8)
Severity of pain					
Mild	12 (20; 10.8 to 32.3)	13 (35.1; 20.2 to 52.5)	1 (12.5; 0.3 to 52.7)	41 (39; 29.7 to 49.1)	67 (32.1; 25.8 to 38.8)
Moderate	45 (75; 62.1 to 85.3)	22 (59.5; 42.1 to 75.2)	4 (50; 15.7 to 84.3)	57 (54.3; 44.3 to 64)	128 (61.2; 54.3 to 67.9)
Severe	3 (5; 10 to 13.9)	2 (5.4; 0.7 to 18.2)	3 (37.5; 8.5 to 75.5)	7 (6.7; 2.7 to 13.3)	14 (6.7; 3.7 to 11)

*FAPD* functional abdominal pain disorders, *FD* functional dyspepsia, *IBS* irritable bowel syndrome, *AM* abdominal migraine, *FAP* functional abdominal pain

### Intestinal and extra-intestinal symptoms in affected children

Loss of appetite (83.3% in FD, 21.3% in control, *p* < 0.001) and flatulence (63.3% in FD, 41.1% in control, *p* < 0.05) were significantly associated with FD while belching (48.6% in IBS, 22.7% in control, *p* < 0.001), vomiting (10.8% in IBS, 2.7% in control, *p* < 0.05), and bloating (21.6% in IBS, 8.4% in control, *p* < 0.05) were more common in adolescents with IBS. Extra-intestinal symptoms were found to be only significantly related to FD with limb pain (51.7% in FD, 35.9% in control, *p* < 0.05) and photophobia (18.3% in FD, 6.3% in control, *p* < 0.01). Light-headedness was found significantly in FD (11.7% in FD, 4.3% in control, *p* < 0.05) and also reported in the AM sub-type (25% in AM, 4.4% in control, *p* < 0.05). The relationships between each symptom to the different FAPD sub-types are shown in Table 4**.**

**Table 4:** Intestinal-related and extra intestinal symptoms in children with functional abdominal pain disorders

	FD, n (%; 95% CI)	IBS, n (%; 95% CI)	AM, n (%; 95% CI)	FAP, n (%; 95% CI)	FGD total, n (%; 95% CI)	Controls, n (%; 95% CI)
Intestinal-related symptoms						
Bloating	9 (15;7.1 to 26.6)	8 (21.6;9.8 to 38.2)*	0	16 (15.2;9 to 23.6)*	33 (15.8;11.1 to 21.5)***	122 (7.7;6.4 to 9)
Loss of appetite	50 (83.3;71.5 to 91.7)***	12 (32.4;18 to 49.8)	7 (87.5;47.3 to 99.7)***	0	68 (32.5;26.2 to 39.3)**	354 (22.1;20.1 to 24.2)
Nausea	0	0	1 (12.5;3 to 52.7)***	0	1 (0.5;0 to 2.6)	10 (0.6;0.3 to 1.1)
Vomiting	1 (1.7;0 to 8.9)	4 (10.8;3 to 25.4)*	4 (50;15.7 to 84.3)***	0	8 (3.8;1.7 to 7.4)	42 (2.7;1.9 to 3.5)
Flatulence	38 (63.3;49.9 to 75.4)*	19 (51.4;34.4 to 68.1)	5 (62.5;24.5 to 91.5)	52 (49.5;39.6 to 59.5)	113 (54.1;47.1 to 61))***	639 (40.3)
Burping	19 (31.7;20.3 to 45)	18 (48.6;31.9 to 65.6)***	4 (50;15.7 to 84.3)	38 (36.2;27 to 46.1)*	78 (37.3;30.7 to 44.3)***	340 (21.4;19.2 to 23.3)
Extra intestinal symptoms						
Headache	4 (6.7;1.8 to 16.2)	1 (2.7; 0.1 to 14.2)	1 (12.5;3 to 52.7)	0	6 (2.9;1.1to 6.1)	37 (2.3;1.6 to 3.2)
Limb pain	31 (51.7;38.4 to 64.8)*	18 (48.6;31.9 to 65.6)	6 (75;34.9 to 96.8)	32 (30.5;21.9 to 40.2)	87 (34.9 to 48.6)	574 (35.9;33.4 to 38.2)
Photophobia	11 (18.3;9.5 to 30.4)**	2 (5.4;0.7 to 18.2)	2 (25;3.2 to 65.1)	0	15 (7.2;4.1 to 11.6)	106 (6.6;5.4 to 7.9)
Light-headed	7 (11.7;4.8 to 22.6)*	2 (5.4;0.7 to 18.2)	2 (25;3.2 to 65.1)*	2 (1.9;0.2 to 6.7)	13 (6.2;3.4 to 10.4)	69 (4.3;3.4 to 5.4)
Fever	1 (1.7; 0 to 8.9)	0	1 (12.5;3 to 52.7)	3 (2.9;0.6 to 8.1)	5 (2.4;0.8 to 5.5)	67 (4.2;3.3 to 5.3)

*AM* abdominal migraine, *FAP* functional abdominal pain, *FD* functional dyspepsia, *FGD* functional gastrointestinal disorders, *IBS* irritable bowel syndrome. *P < 0.05, **P < 0.01, ***P < 0.001, compared with control

### Association between stress and abdominal pain–predominant FAPD

Table 5 shows the association between stressful life-events and FAPD. Divorce or separation of parents (adjusted OR 2.55, 95% CI 1.75–3.7, *p* = < 0.001), death of a close family member (adjusted OR 2.24, 95% CI 1.39–3.59, *P* = 0.001), and father’s alcoholism (adjusted OR 1.94, 95% CI 1.22–3.1, *P* = 0.005) were found to be statistically significant by multivariate analysis in association with FAPD. Since the data collection was conducted in two rounds, a sensitivity analysis was performed for the 2 rounds in collecting methods. The 95% CI adjusted OR for the first and second round of collections were, in the case of divorce or separation of parents 1.41–3.9 and 0.89–3.3; death of a close family member 1.22–4.99 and 0.89–3.3; and of father’s alcoholism 0.98–3.67 and 1.04–3.94. These findings indicate that there is no significant difference in our findings even after comparing the 2 methods of data collection.

**Table 5:** Distribution of responders according to exposure to stressful life events (bivariate and multivariate analysis)

Stressful event	Distribution of subjects		Crude OR (CI 95%)	*p* value	Adjusted OR (CI 95%)	Adjusted *p* value
	Controls *N* = 1.604 *n* (%; 95% CI)	FAPD *N* = 209 n (%; 95% CI)				
Change in school Suspension from school	126 (7.9; 6.6 to 9.3) 170 (10.6; 9.1 to 12.2)	19 (9.1;5.6 to 13.8)) 20 (9.6;5.9 to 14.4)	1.17 (0.7–1.94) 0.89 (0.55–1.45)	0.54 0.64		
Frequent punishment in school	74 (4.6; 3.6 to 5.8)	11 (5.3;2.7 to 9.2)	1.15 (0.6–2.2)	0.68		
Separation from best friend	73 (4.6; 3.5 to 5.7)	15 (7.2;4.1 to 11.6)	1.62 (0.9–2.87)	0.099		
Sitting for government examination	45 (2.8; 2.1 to 3.7)	10 (4.8; 2.3 to 8.6)	1.74 (0.86–3.5)	0.119		
Failure in an examination	46 (2.9; 2.1 to 3.8)	13 (6.2;3.4 to 10.4)	2.24 (1.19–4.22)	0.011		
Being bullied at school	98 (6.1; 5 to 7.4)	20 (9.6;5.9 to 14.4)	1.62 (0.98–2.68)	0.058		
Severe illness in a close family member	108 (6.8; 5.6 to 8.1)	29 (13.9;9.5 to 19.3)	2.22 (1.43–3.44)	< 0.001		
Death of a close family member	88 (8.1; 6.6 to 9.9)	30 (19.5; 13.5 to 26.6)	2.74 (1.74–4.32)	< 0.001	2.24 (1.39–3.59)	0.001
Loss of a parent’s job	667 (41.7; 39.2 to 44)	126 (60.3; 53.3 to 67)	2.13 (1.58–2.85)	< 0.001		
Divorce or separation of parents	291 (18.2;16.3 to 20.1)	82 (39.2;32.6 to 46.2)	2.9 (2.14–3.95)	< 0.001	2.55 (1.76–3.7)	< 0.001
Remarried of parents	155 (9.7; 8,3 to 11.2)	26 (12.4;8.3 to 17.7)	1.33 (0.85–2.06)	0.211		
Birth of a sibling	7 (0.4; 0.2 to 0.9)	5 (2.4; 0.8 to 5.5)	5.57 (1.75–17.71)	0.001		
Frequent domestic fights	696 (43.5;40.9 to 45.9)	118 (56.5;49.4 to 63.3)	1.69 (1.26–2.26)	< 0.001		
Frequent punishment by the parents	494 (30.9;28.5 to 33.1)	98 (46.9;40 to 53.9)	1.98 (1.48–2.65)	< 0.001		
Father’s alcoholism	126 (7.9; 6.6 to 9.3)	36 (17.2;12.4 to 23)	2.43 (1.63–3.64)	< 0.001	1.94 (1.22–3.1)	0.005
Hospitalization of the child for other illness	379 (23.7;21.6 to 25.8)	80 (38.3;31.7 to 45.2)	2 (1.48–2.7)	< 0.001		
Exposure to at least 1 stressful events	322 (20.1;18.1 to 20.1)	47 (22.5;17 to 28.8)	1.15 (0.81–1.63)	0.42		

*CI* confidence interval, *OR* odds ratio, *FAPD* functional abdominal pain disorders

## Discussion

This is the first study from Indonesia to evaluate the prevalence of FAPD in the adolescent population. We found an overall prevalence of 11.5% of FAPD among Indonesian adolescents. FAP (5.8%) was the most prevalent form of FAPD, followed by FD (3.3%), IBS (2%), and AM (0.4%). Overall prevalence of FAPD was higher among females. Most of the gastro-intestinal-related symptoms such as bloating, loss of appetite, belching, and flatulence were significantly associated with FAPD. Stressful life-events such as divorce or separation of parents, death of a close family member and father’s alcoholism were significantly associated with FAPD.

Only few studies have assessed the overall prevalence of FAPD in Asia. Two separate studies from Sri Lanka have reported prevalence rates of 10.8 and 13.8% [1, 14], whereas a lower rate was noted in India (6.2%) [15]. In accordance with our data, other epidemiological studies around the world reported similar overall prevalence rates of FAPD; 12.5% in Colombia, 12.1% in Panama and 9.9% in Nigeria [5, 16, 17]. These findings were different from a study from Jordan, which reported a prevalence of 25.7% [18]. In our study, FAP was the commonest FAPD with a prevalence rate of 5.8% followed by FD (3.3%), IBS (2.0%), and AM (0.4%). In contrast, a study from Sri Lanka reported IBS as the commonest functional abdominal pain disorder (7.0%) whereas the prevalence of FAP, FD and AM were 3.0, 3.5, and 0.2% respectively. An epidemiological survey in Indian adolescents noted FD as the commonest functional abdominal pain disorder (2.7%), followed by AM (1.4%), IBS (1.3%), and FAP (0.3%) [15]. In Nigeria, IBS was found to be the commonest functional abdominal pain disorder (5.6%). However, studies from USA, Greece and the Mediterranean region, reported AM as the commonest functional abdominal pain disorder (9.2, 6.8,7.8% respectively) [19, 20].

The reasons for these differences in prevalence rates are not entirely clear. The aetiology of FAPD is multi-factorial. It is well known that adverse socio-environmental factors, abnormal brain-gut and hypothalamo-pituitary-adrenal axis interactions, and alteration in intestinal microbiota play crucial roles in the pathophysiology of FAPD [21, 22]. Differences in these factors across countries would have contributed, at least partly, to these differences. In addition, inclusion of children/adolescents of different age groups as well as differences in data collection may also have played a part in the differences in these prevalence data. More importantly, subtle differences in translations and cultural differences in understanding and interpreting questions in the QPGS questionnaire may have had a significant role to play in differences in these epidemiological figures.

In accordance with earlier studies a higher female predominance was found in patients with FAPD [1, 18, 23]. The explanation for this finding has not been well established. It has been suggested that female reproductive hormones increase the tendency to develop FAPD [24]. Gender predominance may also be related to the role of visceral hypersensitivity in the pathogenesis of FAPD in children [24, 25]. According to a previous study by Castiloux et al. [26], girls were found to have higher rectal hypersensitivity that leads to manifestation of FAP and IBS than males. For the subtype of FAPD analysis, we only found a significant correlation between FD and female gender (*p* < 0.001), while other sub-types (IBS, AM, and FAP) failed to demonstrate a significant association with the female gender.

We found that the prevalence rate of adolescents with FGD located in the south of Jakarta was lower than other locations in Jakarta. The reason for this is not quietly clear. We speculated that the students from that area are just more tolerant, cheerful or more flexible to stressful events than students from other areas of Jakarta.

Similar to other studies [1, 14], we also compared intestinal related symptoms (bloating, loss of appetite and nausea) and extra intestinal symptoms (such as light headedness, photophobia, and limb pains) of children with FAPD with controls. As expected these symptoms are common among children with FAPDs. The importance of detecting these intestinal related and extra-intestinal symptoms was their contribution to severity of disease and quality of life [27]. Several studies have shown that somatic symptoms (indicated by higher somatisation score) negatively affect the health related quality of life of children with functional gastrointestinal disorders such as IBS and constipation [28, 29].

One of the major factors that influence development and exacerbation of FAPD is psychological stress [1, 5, 11, 25]. Experimental studies suggested involvement of the emotional motor system, for example the amygdala, in inducing visceral hypersensitivity that leads to FAPD [3, 17]. In accordance with a study from Sri Lanka this study clearly shows that home and school-related stressful life events such as divorce or separation of parents death of a close family member and father being an alcoholic were associated with FAPD [1]. Furthermore, a study among Korean adolescents with IBS revealed higher stress scores than controls and the degree of stress had a potential relationship to the development of IBS [30]. However, the individual stressful events were not clearly stated in that study. Our findings showed that only family-related stressors, but not school-related stressors, increases the tendency to develop FAPD. All family-related stressors would undoubtedly put children under stress, especially in an Asian country like Indonesia where the integrity of parental relationships and unity of the family are considered to be a key determinant to the well being of children.

The most important strength of this report is that the study was conducted in a school survey setting, it being principally a community-based study and it being able to unravel the true burden of these disorders in a paediatric community in urban Indonesia, which condition is possibly being under-diagnosed in most of the health facilities across the country. This study was performed with nearly 2000 children from 5 regions in Jakarta (Central, North, West, South, and East Jakarta) that strongly represents Indonesian urban children. However, this study was conducted using two different methods of data collection (questionnaires filled at home and questionnaires filled in school). To confirm that the results of the first round and second round were not different from each other we ran a sensitivity analysis in each group and indeed the results were similar, indicated by the overlapping confidence intervals between the results of each groups. Our study did not assess anxiety and depression in the questionnaires, which may have confounded the association between stressors and FAPD. Although our results showed that adjusted OR for death of close family member and divorce or separation of parents are around 2.0–3.0 and father’s alcoholism is around 1.0–2.0 that may be associated with medium and week association between stressors and FGD [31]. However, the cross sectional design of this study does not allow the determination of time course or inference of causality between FGD and stressors.

Besides that, the results of this study may not be applicable to rural settings, which are also important sectors for Indonesia as a developing country. Another limitation noted in this study was that we did not perform a physical examination or additional investigations to exclude organic causes. Organic causes in a previous study in an Asian setting that could mimic symptoms of FAPD include gastro-oesophageal reflux, urinary tract diseases, antral gastritis and intestinal amoebiasis [32]. However, most of the other epidemiological studies have also not conducted a physical examination or investigations to rule out organic disorders [5, 15, 16, 19, 20].

## Conclusions

We propose that FAPD are important and common health problems in Indonesian adolescents with similar prevalence to most of the Asian countries. FAP is the most prevalent form of FAPD in this group of children. Female gender and exposure to home related stressors predispose adolescents to develop FAPD. In this light, it is imperative to increase awareness, among the medical fraternity that cares for children, about the presence of FAPD in Indonesian children and adolescents in an effort towards improving their overall health.

## Data Availability

The datasets used and/or analysed during the current study are available from the corresponding author on a validated request.

## Abbreviations

AM: Abdominal migraine
FAP: Functional abdominal pain
FAPD: Functional abdominal pain disorders
FD: Functional dyspepsia
IBS: Irritable bowel syndrome
